# ER stress-induced cell death proceeds independently of the TRAIL-R2 signaling axis in pancreatic β cells

**DOI:** 10.1038/s41420-022-00830-y

**Published:** 2022-01-24

**Authors:** Cathrin Hagenlocher, Robin Siebert, Bruno Taschke, Senait Wieske, Angelika Hausser, Markus Rehm

**Affiliations:** 1 University of Stuttgart, Institute of Cell Biology and Immunology, Stuttgart, 70569 Germany; 2 University of Stuttgart, Stuttgart Research Center Systems Biology, Stuttgart, 70569 Germany

**Keywords:** Apoptosis, Stress signalling

## Abstract

Prolonged ER stress and the associated unfolded protein response (UPR) can trigger programmed cell death. Studies in cancer cell lines demonstrated that the intracellular accumulation of TRAIL receptor-2 (TRAIL-R2) and the subsequent activation of caspase-8 contribute significantly to apoptosis induction upon ER stress. While this might motivate therapeutic strategies that promote cancer cell death through ER stress-induced caspase-8 activation, it could also support the unwanted demise of non-cancer cells. Here, we therefore investigated if TRAIL-R2 dependent signaling towards apoptosis can be induced in pancreatic β cells, whose loss by prolonged ER stress is associated with the onset of diabetes. Interestingly, we found that elevated ER stress in these cells does not result in TRAIL-R2 transcriptional induction or elevated protein levels, and that the barely detectable expression of TRAIL-R2 is insufficient to allow TRAIL-induced apoptosis to proceed. Overall, this indicates that apoptotic cell death upon ER stress most likely proceeds independent of TRAIL-R2 in pancreatic β cells. Our findings therefore point to differences in ER stress response and death decision-making between cancer cells and pancreatic β cells and also have implications for future targeted treatment strategies that need to differentiate between ER stress susceptibility of cancer cells and pancreatic β cells.

## Introduction

The endoplasmic reticulum (ER) is the major cellular compartment for protein folding and the location for initiating the transport of most membrane located and secreted proteins. ER protein folding is tightly regulated and supported by numerous chaperones that establish appropriate folding environments and by enzymes acting as folding catalysts [1]. Those enzymes also maintain the proper balance between folding capacity and protein load, therefore ensuring ER homeostasis.

Due to environmental or genetic factors, ER homeostasis can be disturbed leading to accumulation of misfolded proteins which results in ER stress [2]. As an adaptive response, the unfolded protein response (UPR) is activated which aims to reduce protein translational load and to expand the protein folding capacity of the ER [3, 4]. Additionally, misfolded proteins are exported for ER-associated degradation (ERAD) through the ubiquitin proteasome system [5]. Under irresolvable or chronic ER stress conditions the UPR fails to restore homeostasis and instead activates cell death effectors [2, 6, 7].

Cells particularly active in protein production include pancreatic β cells which are specialized for the production and controlled secretion of insulin in order to maintain blood glucose homeostasis [2]. Due to their high secretory activities, β cells experience substantial intrinsic ER stress. Additionally, peripheral insulin resistance and chronic hyperglycemia due to a sedentary lifestyle contribute to prolonged and irresolvable ER stress in β cells. Consequently, this can impair insulin biosynthesis and secretion, finally resulting in β cell failure and manifestation of Diabetes mellitus (DM) [8].

Similarly, cancer cells that rapidly proliferate, often in unfavorable growth environments, face substantial demands for protein production and thus experience ER stress. Indeed, this predisposes cancer cells to become sensitive to treatment strategies that intensify ER stress and thereby induce programmed cell death [9]. Importantly, the loss of β cells and the subsequent onset of diabetes have also been reported to result from cancer therapies. Autoimmune endocrinopathies resembling type I DM have been described as an, albeit rare, consequence of therapies with immune checkpoint inhibitors [10, 11]. Various other anti-cancer therapeutics are known to perturb physiological glycaemic control, causing hyperglycemia and consequentially enhance ER stress in β cells [12]. In line with this, the risk for developing diabetes is significantly increased in cancer survivors [13].

The primary mechanism by which cells die upon unresolved ER stress is apoptotic cell death [14, 15]. Apoptosis execution is triggered by the permeabilisation of the outer mitochondrial membrane, a process governed by the interplay of members of the BCL-2 protein family [16]. Of these, pro-apoptotic members such as BIM, PUMA and NOXA have been implicated as crucial mediators in response to ER stress, being induced transcriptionally and/or activated by MAPK signaling as a consequence of PERK and IRE1 signaling arms of the unfolded protein response [3, 15, 17, 18]. Within the PERK signaling arm, the induction of the transcription factor CHOP contributes substantially to BIM and PUMA transcription [3, 19]. Interestingly, PERK signaling or CHOP likewise can induce the expression of TRAIL-R2 upon ER stress [20–23].

TRAIL-R2 is a cell surface receptor implicated in the initiation of canonical extrinsic apoptosis by its agonist TRAIL, with receptor activation followed by the subsequent activation of initiator caspase-8 and signaling being directed towards the BCL-2 protein family through cleavage of BID [24]. While various studies have suggested that TRAIL-R2 upregulation might contribute to synergistic cell death responses of cancer cells upon combined ER stress and TRAIL treatment, TRAIL-R2 has also been implicated in promoting ER stress-induced apoptosis independent of its role as a cell surface death receptor [22]. Here, TRAIL-R2 accumulates and oligomerises in the ER-Golgi intermediate compartment, and the high local abundance of unfolded proteins might allow for agonistic TRAIL-R2 activation [25].

Since ER stress plays a causative role in the loss of pancreatic β cells and the onset of both type I and II DM [7, 26–28], we here studied to which extent the TRAIL-R2-dependent signaling arm, that so far has only been studied in cancer cells, contributes to β cell death upon ER stress.

## Materials and methods

### Reagents

Thapsigargin and Tunicamycin were purchased from Enzo Life Sciences AG (Lausen, Switzerland). DMSO was obtained from Carl Roth GmbH + Co. KG (Karlsruhe, Germany). Q-VD-Oph (QVD) was purchased from Selleckchem (Houston, TX, USA). IZI1551 (TRAIL) was produced as described before [29]. Cycloheximide (CHX) was obtained from Sigma-Aldrich (St. Louis, Missouri, USA) and Necrostatin-1 was from APExBIO (MA, USA).

### Cell culture

Min6 cells were a kind gift from I. Rustenbeck, University of Braunschweig, Germany and originate from an insulinoma of a transgenic mouse harboring the insulin promotor followed by the SV40 T antigen gene [30]. These cells were grown in Dulbecco’s modified eagle medium with high glucose (DMEM, Thermo Fisher Scientific, Gibco, Waltham, MA, USA) supplemented with 15% FBS (FBS Brazil One, Fetal Bovine Serum, PAN-Biotech, Aidenbach, Germany), 1x GlutaMAX^TM^ Supplement (Thermo Fisher Scientific, Gibco, Waltham, MA, USA) and 71.5 µM 2-mercaptoethanol (Sigma-Aldrich, St. Louis, Missouri, USA).

Ins1E cells were obtained from S. Ullrich, University of Tübingen with authorization from C. Wollheim, Lund University and originate from clonal selection of x-ray induced, transplantable insulinomas [31, 32]. Ins1E cells used in this study additionally harbor human insulin linked with GFP at the C-terminus under control of a Doxycycline inducible promotor. Ins1E cells were grown in Roswell Park Memorial Institute medium (RPMI 1640, Thermo Fisher Scientific, Gibco, Waltham, MA, USA) supplemented with 10% FBS (FBS Brazil One, Fetal Bovine Serum, PAN-Biotech, Aidenbach, Germany), 10 mM Hepes, 1 mM Na-Pyruvate (both Thermo Fisher Scientific, Gibco, Waltham, MA, USA) and 500 µM 2-mercaptoethanol (Sigma-Aldrich, St. Louis, Missouri, USA) with addition of 150 µg/ml Hygromycin and 10 µg/ml Blasticidin (both Carl Roth GmbH + Co. KG, Karlsruhe, Germany). For both cell lines medium was changed every second to third day. L929 cells (ATCC # CCL-1) were grown in Dulbecco’s modified eagle medium with high glucose (DMEM, Thermo Fisher Scientific, Gibco, Waltham, MA, USA) supplemented with 10% FBS (FBS Brazil One, Fetal Bovine Serum, PAN-Biotech, Aidenbach, Germany). All cell lines were regularly tested for mycoplasma infection. Cells were authenticated by STR profiling.

Min6 and Ins1E cells stably expressing human MCL-1 or human TRAILR-2 were generated by lentiviral transduction. Full length human *MCL1* was cloned into pLV-EF1α-IRES-Puro (Addgene plasmid # 85132). Full length human *TNFRSF10B* was cloned into pCW57-MCS1-P2A-MCS2 (Neo) (Addgene plasmid # 89180). For production of lentiviral particles, the respective plasmid was co-transfected together with psPAX2 for viral packaging (Addgene plasmid # 12260) and pCMV-VSV-G for the viral envelope (Addgene plasmid # 8454) into LentiX 293 T cells (Takara Bio, Goteborg, Sweden) using Polyethylenimine (MW 25 K, Polysciences Inc., Warrington, PA, USA). Transduced cells were selected with Puromycin (Applichem GmbH, Darmstadt, Germany) or Geneticin/G418 (Carl Roth GmbH + Co. KG, Karlsruhe, Germany). Cells transduced with empty vectors served as controls. TRAIL-R2 expression was induced using 0.01–1 µg/ml doxycycline (Sigma-Aldrich, St. Louis, Missouri, USA) for at least 6 h.

### Cell death measurements

Cells were seeded 48 h prior to stimulation. For treatment, the stimulus was diluted in cell culture medium containing 1 µM Propidium Iodide (PI, Sigma-Aldrich, St. Louis, Missouri, USA). If the treatment contained Q-VD-OPh (QVD), cells were pre-incubated with QVD for 30 min. Cells were kept in an IncuCyte® S3 Live Cell Analysis System (Essen BioScience, Ann Arbor, USA) at 37 °C and 5% CO_2_. Images at 10× magnification were taken using transmitted light and at 585 nm wavelength (PI emission). At end of the experiment, 0.1 µg/µl Digitonin (SERVA Electrophoresis, Heidelberg, Germany) was added to permeabilize surviving cells. Images were analyzed with the semi-automated IncuCyte® S3 software for cell confluency and PI-positive areas. Cell death over time was calculated as percentage of PI-positive area normalized to cell confluency. These values were further normalized to the maximum PI-positive area after Digitonin treatment.

### Western Blotting and antibodies

Cells were washed in cold PBS and homogenized in lysis buffer (150 mM NaCl, 1 mM EDTA, 20 mM TRIS, 1% Triton X-100, pH = 7.6) with addition of cOmplete^TM^ protease inhibitor cocktail (Roche Diagnostics AG, Basel, Switzerland). Total protein concentrations were quantified by Bradford assay. Equal amounts of proteins were supplemented with 5× Laemmli sample buffer (10% SDS, 312.5 mM Tris pH 6.8, 25% β-mercaptoethanol, 25% glycerine, 0.02% bromophenol blue, all chemicals from Carl Roth, Karlsruhe, Germany) and heated to 95 °C for 5 min. Samples were loaded and separated on Bolt 4–12% Bis-Tris Plus Gels (Invitrogen, CA, USA) at 150 V, 400 mA for 40 min. Proteins were transferred to nitrocellulose membranes using the iBlot 2 gel transfer device (Thermo Fisher Scientific, MA, USA). After 1 h blocking with blocking reagent (Roche Diagnostics, Mannheim, Germany) diluted in TBST (1%) the membranes were incubated with primary antibody (diluted in TBST with 0.5% blocking reagent) overnight. After washing with TBST, membranes were incubated with HRP-coupled secondary antibodies (1:10 000, diluted in TBST with 0.5% blocking reagent) for 1 h at room temperature. Following three further washing steps, membranes were incubated with a HRP substrate (SuperSignal West Dura Extended, Thermo Scientific Pierce Protein Biology, Waltham, MA, USA) and signals were detected with an Amersham Imager 600 (GE Healthcare, Freiburg, Germany GmbH).

The following primary antibodies were used for western blotting: Rat monoclonal BIP (1:500, 76-E6, BioLegend Inc., San Diego, California, USA), mouse monoclonal CHOP/GADD 153 (1:200, B-3, Santa Cruz Biotechnology Inc., Dallas, Texas, U.S.A.), mouse monoclonal Vinculin (1:500, 7F9, Santa Cruz Biotechnology Inc., Dallas, Texas, U.S.A.), rabbit polyclonal TRAIL-R2 (1:500, ab8416, Abcam, Cambridge, UK), rabbit monoclonal TRAIL-R2 (1:800, D4E9, #8074), mouse monoclonal alpha-Tubulin (1:10 000, DM1A, # 3873), rabbit monoclonal MCL-1 (1:1 000, D2W9E, # 94296), rabbit polyclonal Caspase-3 (1:1000, # 9662), rabbit polyclonal PARP (1:1 000, # 9542) all from Cell Signaling (Danvers, MA, USA), mouse monoclonal cleaved PARP (1:700, Asp214, #51–9000017, BD Biosciences, Heidelberg, Germany). The following secondary antibodies were used: Goat anti-mouse (H + L), POD conjugated and goat anti-rabbit (H + L), POD conjugated (both Dianova GmbH, Hamburg, Germany); goat anti-rat (H + L), HRG conjugated (Antibodies-online GmbH, Aachen, Germany).

### qPCR

RNA was extracted using the NucleoSpin® RNA Kit (Macherey-Nagel GmbH & Co. KG, Düren, Germany) following the manufacturer’s instruction. RNA concentrations were measured on a NanoDrop® Spectrophotometer ND-1000 (Thermo Fisher Scientific). Primers were designed with Primer-BLAST from NCBI or chosen from PrimerBank [33] and ordered from Biomers (Ulm, Germany). Mm_Actb_2_SG QuantiTect Primer Assay was ordered from Qiagen (Hilden, Germany). qPCR was performed using the Power SYBR® Green RNA-to-C_t_™ 1-Step Kit with ranges of 0.1 ng to 300 ng of RNA per reaction for standard curve generation (Thermo Fisher Scientific Corporation, Waltham, Massachusetts, USA) following the manufacturer’s instructions. Reactions were measured on a CFX96 Real-Time PCR detection device (Bio-Rad Laboratories GmbH, Hercules, USA). The following mouse qPCR primers were used: *Tnfrsf1a*: 5′- GCTGACCCTCTGCTCTACGA-3′ (forward primer), 5′-TCGCAAGGTCTGCATTGTCA-3′ (reverse primer); *Tnfrsf10b*: 5′-GCAGAGAGGGTATTGACTACAC-3′ (forward primer 1, PrimerBank ID 274319392c2), 5′-GCATCGGGTTTCTACGACTTT-3’ (reverse primer 1, PrimerBank ID 274319392c2), 5′-CGGGCAGATCACTACACCC -3′ (forward primer 2, PrimerBank ID 31981095a1), 5′-TGTTACTGGAACAAAGACAGCC-3′ (reverse primer 2, PrimerBank ID 31981095a1); the following rat qPCR primers were used: *Actb*: 5′- TGGCTCCTAGCACCATGAAG -3′ (forward primer), 5′- AAACGCAGCTCAGTAACAGTC -3′ (reverse primer); *Tnfrsf1a*: 5′- AAGTGCCACAAAGGAACCTAC -3′ (forward primer), 5′- CGTGGTTCTGCGAAGCTGTA -3′ (reverse primer); *Tnfrsf10b*: 5′- CCGGAAGTGTGTCCCCAAAA -3′ (forward primer 1), 5′- TCATGGTCCTGTTCACAGCCT -3′ (reverse primer 1), 5′- CTCACCACAACACGGAACCT -3′ (forward primer 2), 5′- CGAACAGCGCTCGAAGATCA -3′ (reverse primer 2).

### Statistical analysis

GraphPad Prism (GraphPad Software, San Diego, CA, USA) was used for statistical analysis.

## Results

### Thapsigargin and Tunicamycin treatment induce ER stress and dose dependent cell death in pancreatic β cells

**Fig. 1:**
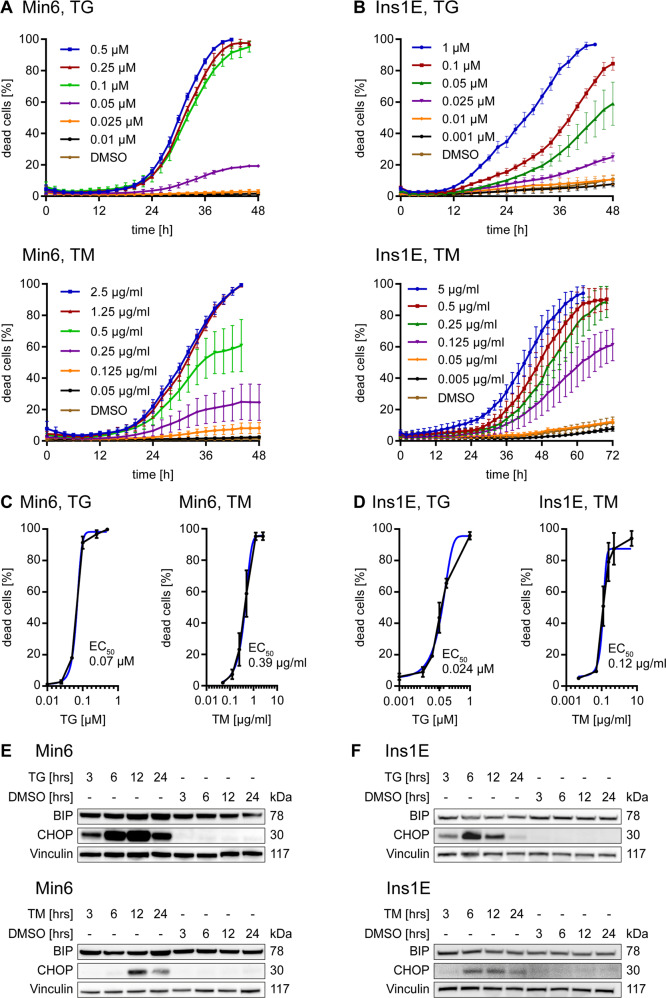
Thapsigargin and Tunicamycin treatment induce robust ER stress and cell death in pancreatic β cells. **A**, **B** Dose-dependent death kinetics of Min6 and Ins1E cells after stimulation with Thapsigargin (TG) or Tunicamycin (TM). Kinetic analyses in high response scenarios were terminated once cell detachment and cell loss made readouts unreliable. All data are from *n* = 3 independent experiments and mean ± SEM. **C**, **D** EC_50_ determination from response data shown in **A** and **B**. EC_50_ values were derived from endpoint readings at 42 h or for TM treated Ins1E cells at 62 h of treatment. **E**, **F** Protein amounts of BIP and CHOP after treatment with TG, TM or DMSO were determined by immunoblotting. Vinculin served as loading control. Min6 cells were treated with 0.15 µM TG or 0.35 µg/ml TM. Ins1E cells were treated with 0.025 µM TG or 0.125 µg/ml TM.

Thapsigargin (TG) and Tunicamycin (TM) are commonly used ER stress inducers which dysregulate ER homeostasis by blocking the ER Ca^2+^ ATPase (SERCA) or inhibiting N-glycosylation respectively [34]. We initially tested if those inducers of pathological ER stress dose dependently induce cell death and associated ER stress markers in pancreatic β cell lines. For this, we employed Min6 and Ins1E cells which are among the most widely used rodent β cell lines since they are two of only a few preserving morphological and physiological characteristics of native β cells [30, 35, 36] (also compare materials section). Most importantly, both retain a differentiated phenotype in cell culture and secret insulin as a response to high extracellular glucose [30, 31] and are therefore considered as a stable and valuable β cell model. Stimulating Min6 and Ins1E cells with TG or TM triggered concentration-dependent cell death in both cell line models, with high concentrations eliminating practically the entire cell populations (Fig. 1A, B). In Min6 cells, lowering the concentrations of TG resulted in a sudden transition towards conditions at which only small fractions of the cell populations died (Fig. 1A), whereas Ins1E cell populations more gradually resisted TG and TM induced stress (Fig. 1B), indicative of higher cell-to-cell response heterogeneities in the latter cell line. Both cell lines thus are susceptible to ER stress, however with differences in their responsiveness. These might be due to differences in species background, cell line origin and development approaches (see materials section).

Irrespective of the drug, cell death initially began to manifest slowly, with notable increases observable only after at least 12 h of treatment. EC_50_ determinations from these data are displayed in Fig. 1C, D. Compared to EC_50_ concentrations for TG and TM treatments reported for cancer cell lines [21, 22, 37, 38], pancreatic β cells appear to be much more sensitive to ER stress-inducing agents, likely reflecting their intrinsically high unfolded protein load resulting from secretory activity [39]. Treated at concentrations close to the EC_50_, the ER resident chaperone BIP, which can accumulate in response to ER stress, only increased in TG-treated Min6 cells (Fig. 1E). On the other hand, the ER-stress induced transcription factor CHOP, which has been linked to TRAIL-R2 induction upon ER stress [21], accumulated strongly in both model cell lines for both treatment scenarios (Fig. 1E, F). Overall, these results demonstrate that pharmacologically induced conditions of elevated ER stress result in the accumulation of a typically cell death-associated transcription factor and pronounced cell death of pancreatic β cells.

### Apoptosis is the primary cell death modality upon ER stress in pancreatic β cells but inhibition of caspases fails to prevent cell death

**Fig. 2:**
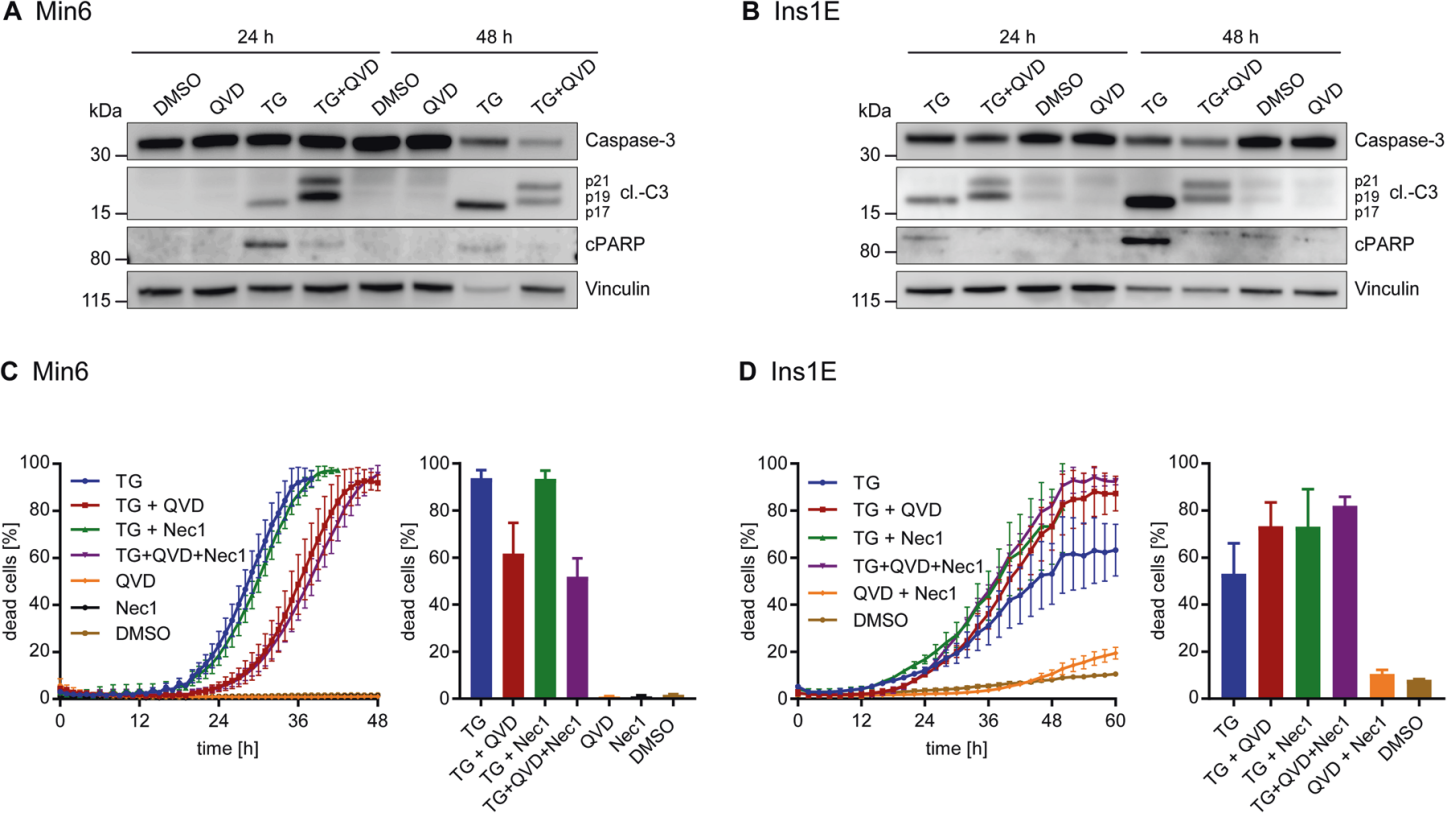
Apoptosis is the primary cell death modality upon ER stress in pancreatic β cells but inhibition of caspases fails to prevent cell death. **A**, **B** Min6 (**A**) or Ins1E cells (**B**) were treated with 0.15 µM TG (**A**) or 0.05 µM TG (**B**), 50 µM QVD, a combination thereof or DMSO for 24 h and 48 h. Protein amounts of procaspase-3, its cleavage products and cleaved PARP were determined by immunoblotting. Vinculin served as loading control. **C**, **D** Cell death measurements in Min6 cells treated with 0.15 µM TG (**C**) or Ins1E cells treated with 0.05 µM TG (**D**), 50 µM QVD, 50 µM Necrostatin-1 (Nec1), a combination thereof or DMSO. PI uptake served as a read-out for cell death. Kinetic analyses were terminated once cell detachment and cell loss made readouts unreliable. Bar graphs display cell death at 38 h (**C**) or 48 h (**D**). Data show means ± SEM from *n* = 3 independent experiments.

We next determined the modality by which pancreatic β cells execute cell death in response to ER stress. Treatment conditions at which TG triggered-ER stress induced cell death in both cell lines correlated with the appearance of fully processed fragments of effector caspase-3, the major executioner caspase during apoptotic cell death (Fig. 2A, B). Similarly, the cleaved 89 kDa fragment of PARP, a hallmark substrate of caspase-3, accumulated upon TG treatment in both cell lines (Fig. 2A, B). In presence of caspase inhibitor Q-VD-Oph (QVD), caspase-3 was incompletely processed to the p21 and p19 subunits, indicating that terminal apoptosis execution was prevented (Fig. 2A, B). Correspondingly, PARP cleavage was no longer detectable (Fig. 2A, B).

However, pan-caspase inhibitor QVD did not prevent but only delay cell death in the Min6 cell populations (Fig. 2C). In contrast, RIPK1 inhibitor Necrostatin-1 (Nec1) failed to protect Min6 cells, suggesting that necroptotic cell death does not contribute to cell death execution (Fig. 2C). Similarly, the combination of QVD and Nec1 conferred no additional protection or delay in cell death (Fig. 2C), suggesting that cells incapable of activating caspases nevertheless die, probably by necrosis. In Ins1E cells, QVD and Nec1, alone or together, failed to delay or prevent cell death upon TG treatment (Fig. 2D), similarly suggesting necrotic cell death if apoptosis cannot be fully executed.

Overall, these findings indicate that apoptosis is the primary form of programmed cell death induced in pancreatic β cells upon persistent ER stress, as evidenced by processing of the primary executioner caspase-3 and cleavage of PARP. However, caspase inhibition ultimately fails to prevent cell death.

### Cell death upon ER stress is induced through the mitochondrial apoptosis pathway

While preventing caspase activation did not avert cell death, we hypothesized that apoptosis signaling nevertheless could be implicated in β cell death upon ER stress. In particular, we set out to test if cell death required the activation of the mitochondrial apoptosis pathway that is regulated by the BCL-2 protein family.

**Fig. 3:**
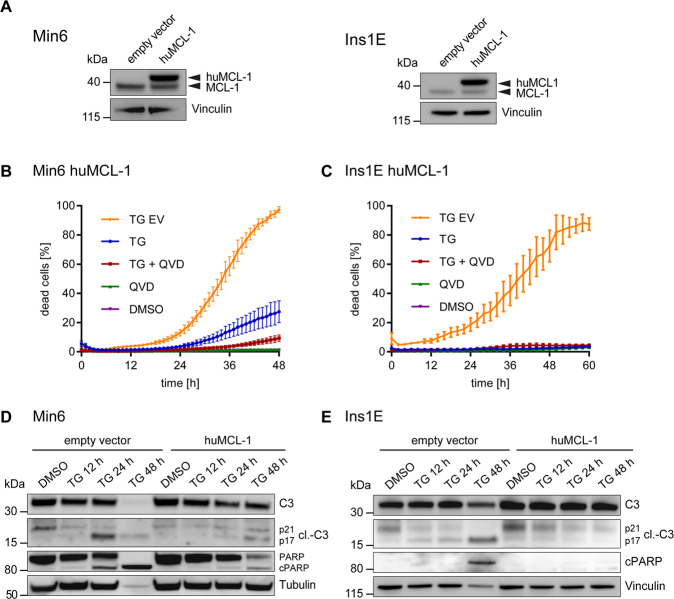
Cell death upon ER stress is induced through the mitochondrial apoptosis pathway. **A** Immunoblotting for MCL-1 in Min6 and Ins1E cells expressing human MCL-1 vs. control cells. Vinculin served as loading control. **B**, **C** Cell death kinetics in Min6 (**B**) and Ins1E cells (**C**) expressing human MCL-1 vs. controls (EV, empty vector). Cells were treated with 0.15 µM TG (**B**) or 0.05 µM TG (**C**), 50 µM QVD, a combination thereof or DMSO. PI uptake served as a read-out for cell death. Data show means ± SEM from *n* = 3 independent experiments. **D**, **E** Immunoblotting for protein amounts of procaspase-3, its cleavage products, PARP and cleaved PARP after TG treatment of Min6 (**D**) or Ins1E cells (**E**) expressing human MCL-1 vs. control cells (empty vector). Cells were treated with same concentrations as above. Tubulin and Vinculin served as loading controls.

To test reliance on mitochondrial apoptosis signaling, we increased the expression of the anti-apoptotic BCL-2 family member MCL-1 in pancreatic β cells (Fig. 3A). Elevating MCL-1 expression indeed potently protected Min6 cells from ER stress-induced cell death, with combined MCL-1 expression and caspase inhibition nearly fully abolishing cell death in these pancreatic β cells across the studied time frame (Fig. 3B). Even more so, MCL-1 expression alone was sufficient to entirely prevent cell death in Ins1E cells (Fig. 3C). Corresponding to these results, increasing MCL-1 expression also suppressed full maturation of caspase-3 in Min6 cells, similar to direct caspase inhibition (Fig. 3D). Accordingly, accumulation of cleaved PARP fragments was reduced (Fig. 3D). Similarly, MCL-1 expression prevented caspase-3 processing and PARP cleavage in Ins1E cells (Fig. 3E). These data therefore indicate that the primary signaling pathway to cell death upon elevated ER stress is the canonical mitochondrial apoptosis pathway.

### Pancreatic β cells express negligible amounts of TRAIL-R2 and resist TRAIL-R2 dependent cell death induction

Due to accumulating evidence for the involvement of TRAIL receptors and the subsequent activation of caspase-8 in ER stress-induced cell death in cancer cells [21, 22, 25], we next studied TRAIL-R2 receptor expression and the competency of pancreatic β cells to commit to TRAIL-R2 dependent cell death.

**Fig. 4:**
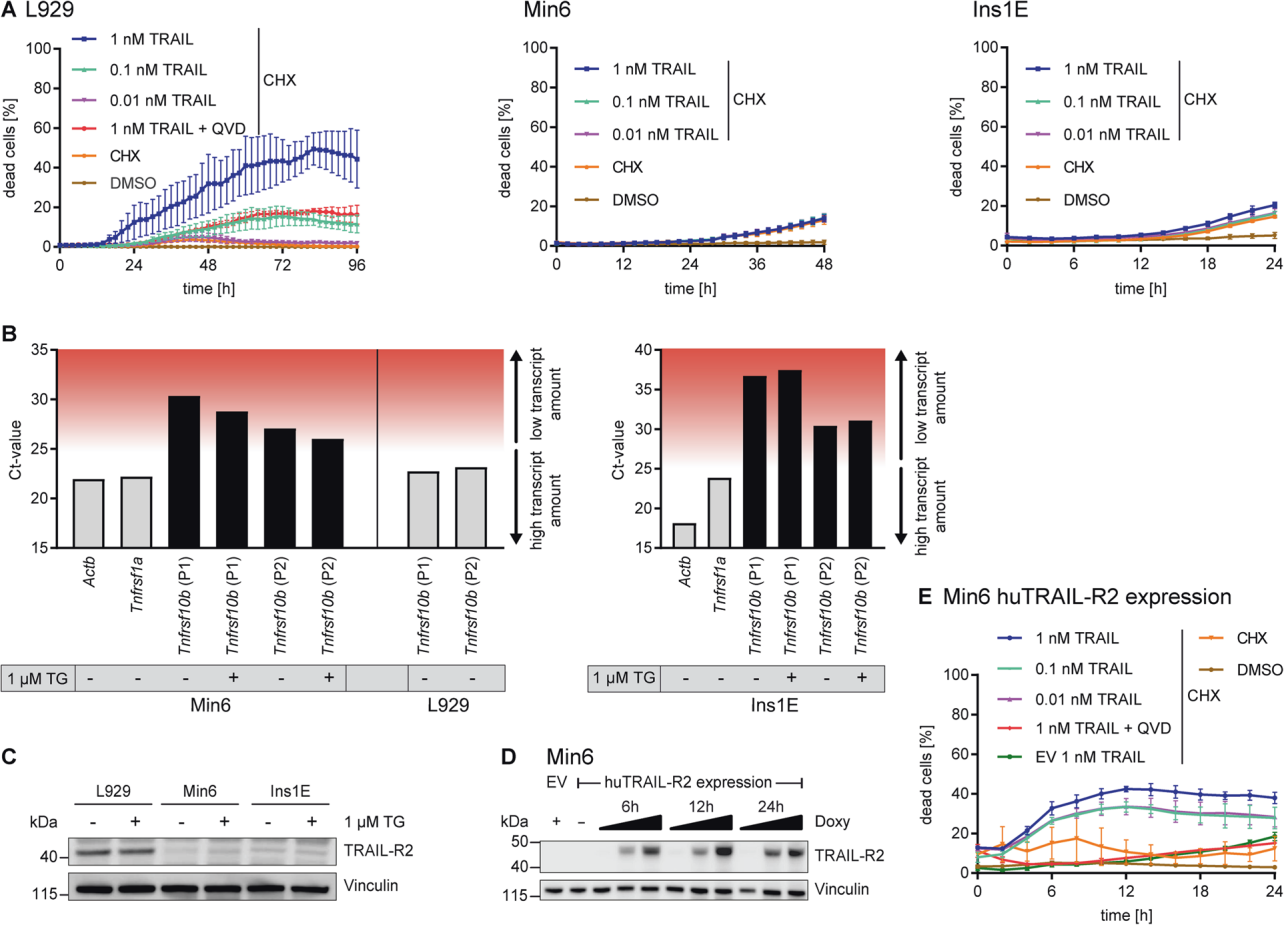
Pancreatic β cells express negligible amounts of TRAIL-R2 and resist TRAIL-R2 dependent cell death induction. **A** Cell-death measurements in L929, Min6 and Ins1E cells treated with hexavalent TRAIL in presence of sensitizer cycloheximide (CHX, 1 µg/ml). PI uptake served as a read-out for cell death. Data show mean ± SEM from *n* = 3 independent experiments. **B** Amplification cycles from qPCR studies including independent *Tnfrsf10b* primer pairs. Displayed are Ct-values of the respective qPCRs on RNA without (−) or with (+) 1 µM TG stimulation for 24 h prior to RNA isolation. Red background indicates increasingly unspecific amplifications. **C** Immunoblot comparison of TRAIL-R2 protein amounts between L929, Min6 and Ins1E cells without (−) or with (+) 1 µM TG stimulation for 24 h prior to protein isolation. **D** Min6 cells expressing doxycycline-inducible human TRAIL-R2 or control cells were stimulated with increasing concentrations of doxycycline. TRAIL-R2 expression was monitored by immunoblotting. EV, empty vector. **E** Cell-death measurements after treatment of Min6 cells expressing human TRAIL-R2 with hexavalent TRAIL, 1 µg/ml cycloheximide (CHX) and 50 µM QVD. PI uptake served as a read-out for cell death. Cells were pre-treated with 0.1 µg/ml doxycycline for 24 h. Shown is one representative graph, mean ± SD of two independent experiments.

To this end, we first tested cell death induction via the extrinsic apoptotic pathway by treating pancreatic β cells with a highly potent hexavalent TRAIL receptor agonist [29] in presence of cycloheximide (CHX). In contrast to TRAIL/CHX responsive mouse fibroblasts (L929), both Min6 and Ins1E cells failed to undergo TRAIL-induced apoptosis (Fig. 4A).

Due to this treatment resistance, we next studied the expression of the TRAIL-R2 coding gene *Tnfrsf10b* in pancreatic β cells. qPCR analyses reproducibly required >25 cycles in Min6 or >30 cycles in Ins1E for product amplification with independent primer pairs, indicative of very low or absent expression of *Tnfrsf10b*, regardless of whether cells experienced ER stress or not (Fig. 4B). In contrast, *Actb* and *Tnfrsf1a* transcripts, coding for beta-actin or TNF receptor 1, accumulated at earlier amplification cycles. Corresponding to response data shown in Fig. 4A, TRAIL responsive L929 cells presented abundant *Tnfrsf10b* transcripts (Fig. 4B), confirming primer specificity and strengthening the overall conclusion that pancreatic β cells express very low amounts of TRAIL-R2. These results corresponded to protein amounts detected by immunoblotting, with Min6 and Ins1E cells expressing substantially lower amounts of TRAIL-R2 than L929 cells (Fig. 4C). Furthermore, TRAIL-R2 did not accumulate upon ER stress (Fig. 4C). Introducing human TRAIL-R2 into Min6 cells rendered these cells sensitive to TRAIL treatment (Fig. 4D, E), indicating that TRAIL resistance can be attributed to insufficient TRAIL-R2 expression but not to dysfunctions downstream in the apoptosis signal transduction cascade.

Overall, these results demonstrate that pancreatic β cells express only negligible amounts of TRAIL-R2 and fail to accumulate TRAIL-R2 upon ER stress. Furthermore, baseline expression of TRAIL-R2 is insufficient to trigger TRAIL-induced apoptosis. Although the signaling cascade downstream of TRAIL-R2 is functional, pancreatic β cells are most likely not capable to engage this signaling arm upon ER stress.

## Discussion

Here, we report that pancreatic β cells respond to elevated ER stress by triggering programmed cell death through the mitochondrial apoptosis pathway that is regulated by BCL-2 family members, however, mostly likely without engaging the TRAIL-R2 dependent signaling arm. This assumption is based on transcript amounts of *Tnfrsf10b* remaining below the threshold for specific detection, barely detectable protein amounts of TRAIL-R2 and lack of accumulation of either TRAIL-R2 transcript or protein upon ER stress.

Permeabilisation of the outer mitochondrial membrane during mitochondrial apoptosis triggers caspase-dependent apoptosis execution and simultaneously results in a bioenergetics crisis due to loss of ATP production capacities by oxidative phosphorylation. As long as cells are incapable of meeting energy demands independent of mitochondrial respiration [40, 41], mitochondrial permeabilisation can be considered a point of no return for cell death even in the absence of caspase activation. Correspondingly, pre-treating β cells with a pan-caspase inhibitor failed to prevent cell death upon ER stress in our hands.

Signaling pathways leading to ER stress and β cell death have been described in the past for both forms of diabetes mellitus (DM). In Type 1 DM, invading immune cells secrete cytokines like TNF-α and IFN-γ which signal via their respective receptors and a NF-κB and STAT-1 dependent signaling network [42]. Of note, TNF-α/TNFR1 activated NF-κB was described as a major survival signal in the TNFR1 pathway [43] and additionally our results showed that TNRF1 is expressed in murine pancreatic β cells. In Type II DM, upregulated insulin production due to a peripheral insulin resistance overloads the ER, resulting in elevated ER stress and UPR activation. Chronic exposure to glucose and free fatty acids (FFA) further enhances this [39].

During ER stress the sensors IRE1 and PERK are activated, which leads to translation of the transcription factor CHOP. The latter has been reported independently by several groups to drive *Tnfrsf10b* expression and intracellular TRAIL-R2 accumulation [22, 23, 44]. Initially, this is counteracted by IRE1 dependent mRNA decay (RIDD) and thus degradation of TRAIL-R2 mRNA. Under prolonged ER stress IRE1 RNase activity is inhibited and therefore maintained PERK signaling overruns RIDD degradation of TRAIL-R2 mRNA, resulting in up-regulation of TRAIL-R2 and apoptosis commitment [4]. The recently proposed binding of TRAIL-R2 to misfolded proteins and subsequent activation of caspase-8 renders TRAIL-R2 a late sensor for misfolded proteins before apoptosis initiation [25].

CHOP-dependent upregulation of TRAIL-R2 with subsequent ligand-independent induction of cell death has been reported not only for several cancer cell lines [21] but also for non-transformed human cells. For example, in hepatocytes, lipoapoptosis was proposed to result from CHOP-dependent transcriptional upregulation of TRAIL-R2 and ligand-independent cell death [45]. In contrast to that, we demonstrated that in pancreatic β cells TRAIL-R2 is barely expressed at baseline and after ER stress, even though the amount of CHOP substantially increased. It has already been shown that ER stress in β cells is initiated and intensified over time through multiple mechanisms, ultimately leading to cell death induction and manifestation of DM [8]. This slow and multi-modular process might have led to adaptive measures in these non-transformed cells, preventing a fast and direct cell death induction upon ER stress.

However, alternative mechanisms of TRAIL-R2 engagement in pancreatic β cells, independent of transcriptional upregulation, may also be possible, albeit unlikely, and therefore cannot be formally excluded based on our results. For example, an upregulation of TRAIL and autocrine TRAIL-R2 engagement upon ER stress might bypass the need for TRAIL-R2 upregulation. However, exogenously added TRAIL failed to induce cell death in our hands. It was previously reported in cancer cells that TG stimulation can lead to upregulation of TRAIL, but also a parallel accumulation of TRAIL-R2 was observed [46]. Furthermore, findings in non-transformed hepatocytes indicated that lipotoxic ER stress solely relies on TRAIL-R2 upregulation and is ligand-independent [45].

Aggregation of the residual amounts of TRAIL-R2 protein in pancreatic β cells could in principle lead to a ligand-independent induction of apoptotic signaling upon ER stress. However, it has been shown that ER stress-induced caspase-8 activation relies on a transcriptional upregulation of *Tnfrsf10b* in cancer cells that express notably higher amounts of TRAIL-R2 [4, 22, 23]. It therefore seems highly unlikely that intracellular aggregation of the low amounts of TRAIL-R2 in pancreatic β cells would contribute meaningfully to the overall cell death response.

Interestingly, TRAIL decoy receptors were reported to be highly expressed in human pancreatic islets [47] and the majority of primary islets and β cell lines seems to be resistant against extrinsic, TRAIL-induced apoptosis [48–50]. These findings already suggest that induction of cell death via the extrinsic TRAIL-R2 apoptosis pathway is avoided in β cells, although functional in principle, as our results showed that enhancement of TRAIL-R2 expression sensitized β cells to TRAIL. In rat β cells, treatment with TRAIL resulted in an activation of NF-κB, which consequently led to upregulation of TRAIL decoy receptors and a simultaneous down-regulation of TRAIL-R2, thereby preventing cell death [49], rendering activated NF-κB as a pro-survival signal, as described for the TNFR1 pathway [43]. Furthermore and in contrast to cancer cells [21, 22], our findings of a low TRAIL-R2 expression even under ER stress conditions indicate that in pancreatic β cells ER stress does not directly signal via an intracellular upregulation of TRAIL-R2. The lack of TRAIL-R2 expression could therefore serve as a regulating mechanism in β cells, which frequently encounter ER stress during periods of high folding demand, to restore homeostasis without causing premature cell death under ER stress conditions. It is tempting to speculate whether this is due to epigenetic modifications such as promotor methylation, leading to the observed lack or low level expression of TRAIL-R2 in murine β cells.

The murine cell lines used in this study are common models to study ER stress and apoptosis in pancreatic β cells [34, 51]. In contrast to human cells, murine cells only express one TRAIL-R orthologue referred to as mTRAIL-R2 [52, 53]. Although showing more similarity to the human TRAIL-R2, some differences in post-translational modifications have already been reported [53, 54]. Although it is questionable whether these differences have a significant impact on apoptosis induction [53, 54], they may limit the comparability of the mechanisms of cell death in murine and human β cells under ER stress. Therefore, it would be of great interest to extend the results shown in this study with work in human β cells in the future [55].

Taken together we have shown in this study that pancreatic β cells express only low levels of endogenous TRAIL-R2 and that persisting ER stress does not result in its transcriptional induction. Also, apoptotic cell death upon persisting ER stress most likely is not TRAIL-R2-dependent. Instead, these cells die upon ER stress via the mitochondrial apoptotic pathway. The low TRAIL-R2 expression might allow β cells a longer adaptation period to ER stress before committing to cell death. Our results suggest that ER stress responses and death decision making differ between cancer and β cells, opening the way for new treatment strategies in cancer therapy: ER stress and cell death initiation can be targeted to eliminate cancer cells while preserving normal β cell function. This would render the TRAIL-R2 signaling pathway a suitable target in cancer therapy while reducing the risk of diabetes development in cancer survivors.

## Data Availability

The data generated and/or analyzed during the current study are available from the corresponding author on reasonable request.
